# Vocational Rehabilitation of Young Adults with Psychological Disabilities

**DOI:** 10.1007/s10926-018-9773-y

**Published:** 2018-05-10

**Authors:** Silke Tophoven, Nancy Reims, Anita Tisch

**Affiliations:** 1 Institute for Employment Research (IAB), Regensburger Strasse 104, 90478 Nuremberg, Germany; 2 City of Krefeld, 47792 Krefeld, Germany; 3 Federal Institute for Occupational Safety and Health (BAuA), Friedrich-Henkel-Weg 1-25, 44149 Dortmund, Germany

**Keywords:** Youth, Psychological disabilities, Multi-method approach, Germany

## Abstract

*Objective* Vocational rehabilitation measures support youth and young adults with disabilities to obtain vocational training and to enter the labor market. In Germany, a growing number of young people with psychological disabilities in vocational rehabilitation can be observed. The study at hand focuses on this group and examines their (un-)unemployment biographies before vocational rehabilitation, their access to vocational rehabilitation and identifies their individual challenges within the process of vocational rehabilitation. *Methods* Using a multi-methods approach, we analyze representative administrative data of the German Federal Employment Agency as well as biographical interviews conducted with young rehabilitants. We compare the population of young rehabilitants with psychological disorders to those with other disabilities in terms of vocational rehabilitation and initial labor market entry in order to get a representative picture about their school to work transitions. Since rehabilitants with psychological disabilities tend to be older than the remaining population, analyses are stratified by age groups. In addition, qualitative in-depth interviews provide an additional and deeper understanding of specific employment barriers youth with psychological disorders have to overcome. Furthermore, the individual perspective gives insight on how the crucial transition from school to work is perceived by the population under study. *Results* The pathway into vocational rehabilitation of youth with psychological disorders is often characterized by obstacles in their transition from school to work. During rehabilitation, it appears essential to provide psychological stabilization along with vocational training. Although their average level of education is higher than those of other rehabilitants, labor market transition after (often company-external) vocational training challenges many young people with psychological disabilities, leaving many of them with comparatively poor labor market prospects. *Conclusions* Young persons with psychological disabilities, who come from regular schools or dropped out from regular school or university, seem to find their way to vocational rehabilitation more indirectly. Furthermore, vocational rehabilitation itself is often prolonged for those with psychological disabilities possibly due to a corresponding stabilization process. However, vocational rehabilitation can be a core element within the stabilization process of a psychological disease.

## Introduction

Employment is of primary importance to financial security and social well-being in Western societies [1]. There is no doubt that those with poor health are generally worse off in the labor market. Poor health leads to significantly lower propensities to be employed [2, 3] and unemployment further deteriorates well-being and psychological health [4, 5]. Furthermore, youth with psychological disabilities have difficult school to work transitions and experience unemployment more often [6]. Baron and Salzer discuss three factors explaining the comparatively low labor market participation rates of people with poor psychological health [7]: (a) the illness itself, since the disability is considered too severe to permit work; (b) employer discrimination; and (c) the unavailability of appropriate vocational rehabilitation programs and access to those programs.

In our exploratory study, we focus on (a) and (c) and analyze rehabilitation programs for youth and young adults with psychological disabilities and their transition to the labor market. Vocational rehabilitation aims at improving employment prospects after a phase of incapacity to work and for people with disabilities. Furthermore, in Germany, vocational rehabilitation is intended not only to help people to return to work, but also to help young people in their initial labor market entry.^1^ We focus on measures used to improve the employment prospects of young rehabilitants with psychological disabilities by providing participants with vocational guidance and training.

Transition from youth to adulthood is associated with several challenges. During adolescence, young people are expected to graduate from school, complete vocational training or university, and find their first job and, in addition, move into their first own apartment, have partnerships and start a family. For vulnerable groups, e.g., adolescents with disabilities, these challenges are even more difficult, because they also have to manage limitations due to their disability [8, 9]. Adolescents with mental disorders are particularly vulnerable as they often drop out of school, have a greater likelihood of behavioral problems, and are more likely to enter parenthood early and experience less stable personal relationships [10]. Furthermore, they are prone to be affected repeatedly by mental disorders and setbacks during their life [11]. Their employment biographies are often filled with low-status jobs. Thus, the negative consequences of mental disorders during adolescence often persist in adult life in economic, social and psychological terms [11].

In Germany, the Federal Employment Agency (FEA) is responsible for the initial labor market integration of individuals with disabilities. According to Sect. 2(1) of Book IX of the German Social Code,

a person has a disability if their physical ability, mental capacity or mental health is likely to fall short of that typical for their age for at least six months and their participation in society is consequently impaired. They are deemed at risk from disability if such impairment is anticipated.

Individuals with disabilities or at risk of disability are legally entitled to apply for vocational rehabilitation. In order to support youth with disabilities during their school to work transition, vocational rehabilitation provide participants with career orientation and vocational training.

Pathways to vocational rehabilitation are highly institutionalized for students at special schools. About 1 year before graduation, the rehabilitation personnel of the local employment agency (LEA) visit special schools and provide information on vocational rehabilitation opportunities and support for vocational preparation and training.^2^ However, youth with psychological disabilities often attend mainstream schools, especially when the disease occurs at an older age, e.g., during adolescences, where career counseling is not as institutionalized.

Nevertheless, in Germany over the last decade, rehabilitation requests from youth with psychological disabilities have increased. While youth with learning disabilities remains the largest group among young rehabilitants (about 50%), the share of rehabilitation participants with psychological disorders increased from 15% in 2009 to over 20% in 2014 [13].

The increasing share of youth with psychological disorders is not surprising. Over recent decades, psychological diseases have been increasingly diagnosed, the public has become more aware of psychological disorders, and social acceptance increased [14]. The notion of “psychological disorders” covers a wide range of diseases including schizophrenia, neurotic disorders, depression and addiction [15]. In addition, different diagnoses can occur in parallel and psychological diseases often arise in combination with other disabilities [16–18]. The resulting multi-morbidity entails particular challenges for vocational rehabilitation institutions.

Mental disorders and psychosocial impairments often occur in youth. The episodic and persistent courses of most psychological diseases particularly challenge those affected in terms of their labor market integration [9]. Therefore, special training institutions for youth with psychological disabilities have been established. These institutions offer psychological and pedagogical support along with career orientation and training. Some institutions are organized as residential training centers and focus on the stabilization and qualification of youth with psychological disorders. Targeted measures aim to improve employment prospects and provide participants with vocational training and the ability to cope with their psychological disorder in future working life.

In the study at hand we analyze the specifics of the vocational rehabilitation process experienced by young people with psychological disabilities. We focus on those in training measures and apply a multi-methods approach. An analysis of the FEA’s administrative data allows us to provide a representative picture of individuals with psychological disorders, their entries to vocational rehabilitation and to illness-specific labor market programs. Using representative data, we are able to identify general institutional circumstances and obstacles young men and women with psychological disabilities are confronted with during initial labor market integration. Contrasting their typical situation with those of men and women with other disabilities might indicate whether or not appropriate rehabilitation measures are available or allocated to the target group [19].

Furthermore, we use qualitative in-depth interviews in order to detect potential explanations for differences found in the quantitative analysis and to give initial advice on how future programs must be designed to best suit the particular situation of youth and young adults with psychological disabilities.

Explorative interviews conducted with the target group emphasize the individual perspective of these young rehabilitants with psychological disabilities, their reflections on access to vocational rehabilitation and their experiences during the process of vocational rehabilitation.

Though, there are some studies particularly examining persons with psychological disabilities [20], most studies investigating the labor market situation of persons with psychological disabilities focus on adults [21–25].The few studies of young people with disabilities contrast them and their labor market transition to persons without disabilities [11, 26, 27]. A combination of qualitative and quantitative methods, as we use it, has not been found so far. At least for the German context, the specific perspective of young rehabilitants with psychological disabilities on their employment transition is still under-researched.

Our exploratory inquiry focuses on the following research questions.

1. Based on administrative data, we ask, how the group of young rehabilitants with psychological disorders differs from rehabilitants with other disabilities in terms of their sociodemographic structure and typical rehabilitation process patterns.

Sociodemographic differences as well as extended periods of transition and rehabilitation duration might suggest illness-specific needs and the unavailability of appropriate rehabilitation programs in accordance with Baron and Salzer [7].

2. Thus, we further ask why rehabilitation patterns differ for those with psychological disabilities. Based on qualitative interviews, we therefore generate further hypotheses on how young people with psychological disorders perceive the process of vocational rehabilitation, which typical obstacles they have to overcome before entering vocational rehabilitation and during the process.

## Data and Methods

In order to analyze the specific situation of youth with psychological disabilities in training measures for initial labor market integration, we make use of different data sources and follow diverse research approaches. First, we use consolidated administrative data collected in the vocational rehabilitation processes covered by the FEA (German Reha-Process Data Panel (RehaPro)). The data is comprised of all individuals with periods of vocational rehabilitation covered by the FEA between 2007 and 2014 (n = 354,436) (cf. Table 2 for a description of the study population from the administrative data source). RehaPro includes demographic characteristics such as sex, age and type of disability officially registered, as well as information on periods of education, employment, unemployment and benefits receipt on a daily basis. It covers periods before, after and during vocational rehabilitation. The administrative data not only allows to give a comprehensive and representative description of the population under study, but also to identify changes and trends over time. Using linear regression, we identify group differences between rehabilitants with different types of disability. These differences are characterized in terms of time in unemployment before the take-up of rehabilitation and rehabilitation process times (process times from the LEA’s acceptance of rehabilitation status to the take-up of the first labor market program, the duration of vocational training and the duration of rehabilitation). We use STATA v14.2 for our analyses.

Unfortunately, administrative data is restricted in terms of individual health trajectories, individual experiences, personal reflections and decision paths. Consequently, qualitative, in-depth interviews [28] with 16 individuals with psychological disabilities between the age of 17 and 25 having been assigned to initial labor market integration programs were conducted. On the basis of parameters obtained from quantitative analyses of the administrative data, we were able to draw a sample for the qualitative analyses and then follow the principles of theoretical sampling [29, 30]. In order to ensure symbolic representation [31], women and men from different regions were taken into account, as well as individuals with different educational backgrounds. Furthermore, we focused on vocational rehabilitation measures leading to a recognized qualification (vocational training) and career orientation schemes, taking account of different educational service providers. In semi-structured narrative interviews,^3^ interviewees were asked about how their sickness evolved and how they entered vocational rehabilitation. In order to discover individual experiences and perspectives, we apply an explanatory research strategy [29], allowing individuals to evaluate their own actions. This interview technique allows us to obtain a deeper insight into dynamic processes of vocational rehabilitation. Amongst other topics, they were asked about their participation in labor market programs and their experiences with counselling personnel, training institutions and companies during vocational rehabilitation. Interviews took about 1 to 2 h.

The study population for the qualitative in-depth interviews is described in Table 1. For 12 of the 16 rehabilitants with a psychological disability, the psychological disability is their documented main disability and, therefore, the disability that hinders labor market participation the most. For 4 rehabilitants a learning disability is documented as their main disability and psychological disorders occur as a secondary impairment. In particular, the qualitative interviews give insight into individual social contexts, explanatory approaches and personal problems of the persons concerned. At the time of the interview, for 15 out of the 16 interviewees the vocational rehabilitation process was still ongoing. Nine of the interviewees were participating in a preparation measure and six were attending a vocational training measure. The age of the interviewees ranges from 17 to 25. The sample comprises persons who graduated from special school as well as those with a university entrance qualification. Six rehabilitants report a school dropout when they were asked about their educational level. We use MAXQDA 10 to organize and analyze the qualitative data.

**Table 1:** Study population from the qualitative sample, people in first integration with psychological disabilities Reproduced with permission from Qualitative in-depth interviews with people in first integration 2014/2015

Sample of youth assigned to initial integration programs with psychological disabilities	n = 16
Psychological disability (main disability)/psychological disability (further disability)	n = 12/n = 4
Rehabilitation ongoing/finished	n = 15/n = 1
Preparation measure/vocational training measure	n = 9/n = 7
Male/female	n = 10/n = 6
Age (at time of the interview)	Age ranges from 17 to 25
Educational level or time of dropout from school	University entrance qualification (n = 2), higher secondary school certificate (n = 1) Lower secondary school certificate (n = 3) Special school (n = 3) Dropout from school before completing university entrance qualification (n = 5) Drop out from lower secondary school (n = 1) No information (n = 1)

## Results

First, results based on administrative data are presented. Beside a description of the group of young rehabilitants with psychological disorders in contrast to rehabilitants with other disabilities in terms of their sociodemographic structure, we thereby focus on important biographical steps in the school to work transition of rehabilitants: (1) the transition from school to rehabilitation: the period after the end of schooling, the entry towards vocational rehabilitation and the entry towards the first rehabilitation program after being acknowledged as a rehabilitant, (2) the rehabilitation phase itself: the duration of vocational rehabilitation and vocational training (incl. the type of vocational training) and the labor market entry. In order to account for descriptive differences, we stratify multivariate analyses estimating the two process periods before and during vocational rehabilitation by age groups (younger than 17 years; 17–20 years of age; 21–24 years of age and 25 years or older measured at the start of vocational rehabilitation). Afterwards, results of the qualitative analysis are presented to provide an individual perspective on psychological disorders and educational biographies and to gain a deeper understanding of how different obstacles are perceived by the population studied for the different steps of the process.

Table 2 describes the study population based on the administrative data source at baseline. Individuals with psychological disabilities are compared to other rehabilitants. The average age at the start of rehabilitation is 21 among those with psychological disabilities and 18 among those with other disabilities. Those with psychological disabilities show higher levels of education. In terms of status before entering vocational rehabilitation, young persons with psychological disabilities most often come from unemployment, participated in career counseling or in vocational orientation measures and less often transfer to vocational rehabilitation directly after school attendance in comparison to those with other disabilities (see Table 2). In comparison to other rehabilitants, those with psychological disabilities are longer unemployed before starting vocational rehabilitation (on average 210.07 vs. 69.24 days) and enter rehabilitation later (on average 1329.05 vs. 360.80 days between the end of schooling). However, if rehabilitation starts, the start of the first labor market program commences about two months after being acknowledged as a rehabilitant (78.04 vs. 73.49 days between the start of vocational rehabilitation and the start of the first program). Furthermore, both the rehabilitation process and the duration of vocational training (for those taking up vocational training) are shorter for persons with a psychological disability (719.51 vs. 842.43 days (duration of vocational rehabilitation); 815.50 vs. 986.69 days (duration of vocational training)) (without illustration).

**Table 2:** Study population from the administrative data source: rehabilitants in initial labor market integration programs (starting in 2007 to 2014)—individuals with psychological disabilities in comparison with the other rehabilitants RehaPro, rehabilitation cohort starting in 2007 to 2014, own calculations

Characteristics	In %	
	With a psychological disability (n = 58,238)	Other rehabilitants^a^ (n = 296,198)
Male/female	60/40	61/39
Age		
Under the age of 17	10	26
17–20	43	61
21–24	30	11
25 and older	17	3
Mean (std. dev.)	21.1 (4.72)	18.19 (2.98)
Educational level		
No school leaving certificate	2	2
Graduation from special school	16	21
Lower secondary school certificate	7	32
Higher secondary school certificate	42	38
University entrance qualification	24	6
No information	9	1
Status immediately before vocational rehabilitation		
School	18	55
Career counseling/orientation measures	33	22
Unemployment	35	17
Employment	6	5
No prior information	6	2

^a^In addition to 16.4% persons with psychological disabilities, our analysis population from the administrative data comprises 83.6% persons with other disabilities who can be distinguished into people with a learning disability (55.2%), a mental disability (16.0%), a musculoskeletal disability (4.4%) and different other disability groups (8.0%)

### Quantitative Results I: Transition from School to Rehabilitation

In comparison to youth with other disabilities, those with psychological disabilities show longer periods of unemployment after the end of school. However, stratified analyses by age groups reveal that the result is only valid for those under the age of 20 (Table 3, model 1). Among those between the age of 21 and 25 individuals with learning disabilities show significantly longer periods of unemployment after school, while unemployment duration is shorter among those with mental or musculoskeletal disabilities. In the oldest age group, people with psychological disabilities do not differ from people with mental or musculoskeletal disabilities, while those with learning disabilities experience longer periods of unemployment.

**Table 3:** Linear regression analysis on different process durations Reproduced with permission from RehaPro, own calculations

Stratified according to age (at rehabilitation start)	Model 1				Model 2				Model 3			
	Duration of unemployment before start of rehabilitation				Duration between the end of school and start of rehabilitation				Duration between the start of rehabilitation and start of first labor market measures			
	< 17 years	17–20 years	21–24 years	25 years and older	< 17 years	17–20 years	21–24 years	25 years and older	< 17 years	17–20 years	21–24 years	25 years and older
	b/se	b/se	b/se	b/se	b/se	b/se	b/se	b/se	b/se	b/se	b/se	b/se
Type of disability (ref.: psychological)												
Learning	− 1.25**	− 11.18***	18.16***	46.39*	− 6.99***	− 116.7***	− 60.99***	− 559.25***	12.11***	− 4.84***	− 10.71***	− 7.61*
	0.48	0.77	3.9	19.24	1.27	2.98	12.13	72.89	1.62	0.75	1.39	3.18
Mental	− 2.25*	− 25.88***	− 155.94***	− 7.34	− 13.86***	− 156.38***	− 550.91***	856.94***	− 12.77**	− 8.28***	− 11.2***	− 8.66*
	1.02	0.96	6.3	21.62	2.51	3.41	17.76	85.76	4.02	1.06	2.2	3.4
Musculo-skeletal	− 1.9*	− 14.87***	− 35.08***	− 2.36	− 4.85*	− 79.47***	− 83.52***	173.18+	− 4.3	− 1.76	2.97	6.25
	0.87	1.29	6.3	23.75	2.21	4.78	19.44	90.23	2.91	1.22	2.19	4.12
Constant	4.42***	24.61***	197.49***	189.36*	7.65***	129.7***	590.36***	− 956.23***	73.55***	82.62***	66.77***	66.07***
	0.59	1.15	9.56	93.26	1.57	4.24	25.15	272	2.2	1.3	3.77	15.01
Number of cases	82,046	201,971	47,149	15,001	50,883	108,248	23,017	7512	73,711	181,885	40,835	12,413
R2	0.034	0.183	0.218	0.15	0.362	0.507	0.54	0.125	0.097	0.09	0.056	0.038

Additionally controlled for sex, education, status on entering rehabilitation, year rehabilitation begins (2007 to 2014) and other types of disabilities with low representation within the population

*b* beta coefficients, *se* standard error

*p < 0.05, **p < 0.01, ***p < 0.001; 95% confidence interval

Generally, the period between the end of school and the start of the rehabilitation process is longest for people with psychological disabilities. Only among those at the age of 25 and older, schooling time has passed considerable longer among persons with a mental disability (Table 3, model 2).

If persons are acknowledged as a rehabilitant, it generally takes about two months until the first program starts. Persons with psychological disabilities do not differ from persons with musculoskeletal disabilities in this context over different age groups, but they participate in programs a few days later than persons with mental disabilities (all age groups) and persons with a learning disability (in all age groups except for those younger than 17 years) (Table 3, model 3).

### Quantitative Results II: Rehabilitation Phase

Depending on placement strategies and the state of health of the rehabilitant, the duration of vocational rehabilitation can differ among rehabilitants. While controlling for rehabilitation-specific, structural and employment-specific characteristics, the results show that the mean duration of vocational *rehabilitation* of people with psychological disability is significantly higher than the duration of individuals with learning, mental or musculoskeletal disabilities. This applies for almost all age groups except for persons younger than 17 years of age where persons with a learning disability stay slightly longer in vocational rehabilitation (Table 4, model 4).

**Table 4:** Linear regression analysis on process durations—duration of rehabilitation and vocational training during rehabilitation Reproduced with permission from RehaPro, own calculations

Stratified according to age (at rehabilitation start)	Model 4				Model 5			
	Duration of vocational rehabilitation				Duration of vocational training			
	< 17 years	17–20 years	21–24 years	25 years and older	< 17 years	17–20 years	21–24 years	25 years and older
	b/se	b/se	b/se	b/se	b/se	b/se	b/se	b/se
Type of disability (ref.: psychological)								
Learning	32***	− 13.42**	− 70.49***	− 76.32***	127.43***	115***	79.63***	− 3.79
	8.99	4.28	7.33	14.35	18.17	9.88	16.49	40.15
Mental	− 62.92**	− 91.94***	− 103.22***	− 55.63***				
	19.43	5.78	11.56	15.38				
Musculo-skeletal	− 118.12***	− 149.32***	− 112.51***	− 123.81***	− 78.62**	− 89.38***	− 29.46	− 95.88+
	15.57	6.86	11.49	17.72	28.03	14.47	23.27	53
Constant	515.84***	713.61***	759.54***	738.9***	502.6***	533.89***	745.95***	619.08**
	15.39	7.33	17.72	61.18	24.02	16.61	88.52	235.46
Number of cases	44,128	115,184	26,878	8409	25,786	46,936	10,075	1647
R2	0.534	0.493	0.405	0.346	0.249	0.258	0.201	0.188

Additionally controlled for sex, education, status on entering rehabilitation, year rehabilitation ends (2010 to 2014), other types of disabilities with low representation within the population, main labor market measure/strategy (only model 4), reason for completing rehabilitation (models 4 and 5), type of vocational training (only model 5)

*b* beta coefficients, *se* standard error

*p < 0.05, **p < 0.01, ***p < 0.001; 95% confidence interval; people with mental disabilities have to be included in the category “other disabilities”, since very few of them undertake vocational training

Successful graduation from vocational training is a major factor in obtaining decent employment in Germany [32] and is the most frequently provided measure in vocational rehabilitation [13]. Providing youth with a vocational qualification aims at improving their employability and employment prospects. Almost 40% of young people with psychological disabilities attend vocational training programs during rehabilitation. Usually, vocational training is intended to last between 2 and 3 years. On average, rehabilitants with psychological disabilities take more time for their training than persons with a musculoskeletal disability, but less time than people with learning disabilities. This applies for all age groups except for the age group “older than 25 years” where there are no significant differences between persons with different disabilities (see Table 4, model 5).

There are different types of vocational training opportunities depending on personal needs and skills, as well as on regional supply. Some rehabilitants obtain regular in-company vocational training (with or without subsidies from the FEA), whereas others are provided with vocational training in special vocational training centers with different degrees of in-company experience. German vocational training generally comprises a theoretical and practical part. Rehabilitants attaining in-company vocational training complete the theoretical part at regular vocational schools; the practical part is completed within the company providing the apprenticeship. This type of training corresponds to the regular form of vocational training within the German dual system.

**Fig. 1:**
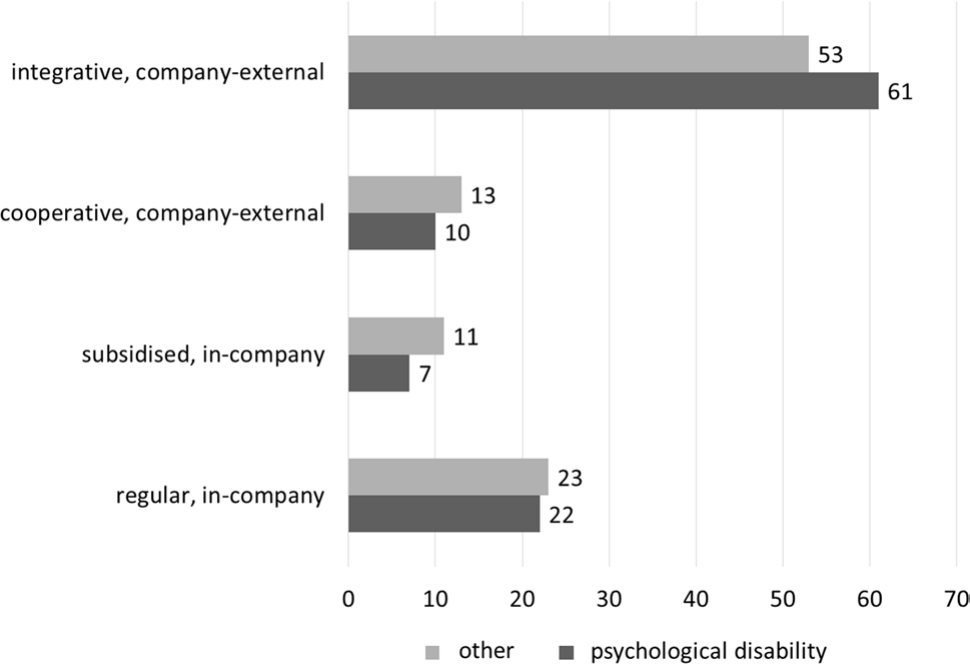
Type of vocational training during vocational rehabilitation, in %. Rehabilitation cohorts ending in 2007–2014, own calculations based on RehaPro

However, most rehabilitants participate in vocational training at rehabilitation-specific and company-external vocational training centers, where theoretical and practical training is provided at the vocational training center (integrative training model) or where the practical part of the vocational training is provided in cooperation with a company (cooperative training model). Integrative training is the most common type of vocational training provided under vocational rehabilitation, particularly among youth with psychological disabilities (see Fig. 1).

Generally, the FEA considers the process of vocational rehabilitation as completed if stable employment, defined as employment for more than 6 months within half a year, is obtained. If a rehabilitant does not enter employment, other outcomes or reasons for ending rehabilitation are noted by the rehabilitation personnel—most commonly sickness or a lack of cooperation. In comparison with rehabilitants as a whole, individuals with psychological disabilities have a considerably lower success rate in obtaining employment (16 vs. 25% in the remaining population). Furthermore, they are more likely to end rehabilitation due to medical reasons. As many as 20% of rehabilitants with psychological disabilities exit vocational rehabilitation due to medical issues (compared to 4% in the remaining population) (without illustration).

### Qualitative Results I: Transition from School to Rehabilitation with Psychological Disabilities

The aim of the qualitative analysis is to generate hypotheses on reasons and explanations for the quantitatively outlined differences between young rehabilitants with psychological disabilities and other young rehabilitants. At the beginning of each interview the interviewer asked the interviewees to describe their access to rehabilitation and their experiences during schooling. The descriptions of the transition phase from school to rehabilitation suggest that most obstacles to vocational education and employment are rooted in their psychological condition. Accordingly, the interviewees reflect on the beginning of their psychological disorder, the c course it has taken. They describe that they struggled at school and dropped out before graduation:

“In the eleventh and twelfth grade I wasn’t able to focus on my school stuff, ... and so then I thought I needed help from someone outside and I first went to hospital, but they couldn’t help me so much there, I was there six, eight weeks and then I went back to school and after about six months in twelfth grade, because I was failing [at school] because of my illness, I ended up in hospital” (EEG_L_b)

The interviewees associate pressure experienced at school as well as during periods of transition between different schools and/or (pre-)vocational training and unsuccessful attempts to find vocational training or employment, difficult family situations and typical problems during puberty with the occurrence of their psychological disorders. Psychological conditions and challenges in the school to work transition can be interpreted as mutually reinforcing and lead to long periods of inactivity:

“... and then I tried to find an apprenticeship [i.e. vocational training] and that didn’t work at all and by the time I left school I already had exam anxiety and such like and that was all made worse by the rejections; and that’s when my social phobia really took hold ... for four years I didn’t really do anything because I didn’t even try and I was also in therapy...” (EEG_M_d)

Interviewees also reflect on their previous biographies and medical histories, explaining how their illnesses dragged them down. The description of a 25-year old man with a gambling addiction illustrates the association between the course of psychological disorder and labor market integration process over a long period for those attending mainstream schools on a higher educational level. He summarizes his personal collapse and his several attempts to find vocational training and employment:

“And at some time (while I was at school) I started going regularly to the amusement arcade... I was pretty addicted. …And then I felt so bad, I didn’t go to school at all. … At school they knew that I was addicted to the machines, and I’d already repeated a year and then despite that they offered me the chance to do another year again. But then, I thought, it’s not really worth it because my grades are so bad. And that’s why I thought, I’ll get an apprenticeship [i.e. vocational training]. … So, I started this training ... but it wasn’t a good time for me, I wasn’t really part of the team, I had difficulties communicating and I was still in the amusement arcade all the time, my wages all went on the machines ... I got through the trial period, but after four months I quit my training, did a bit of casual work and then I wanted to get my high school diploma [Abitur] at the technical high school [Fachoberschule], so then I went to high school ... but I didn’t want to admit to myself that I was in a really bad state. … I could hardly concentrate. So, of course I didn’t get through the trial period at high school. Then I took a mini-job. … But this mini-job wasn’t enough for me and so I wanted to start another apprenticeship. Then I tried to do the training at the same time as my mini-job. And that didn’t work either. And then things just got worse and worse. At some point, I couldn’t even get myself to work anymore... And there I was - simply at home.” (EEG_L_d)

However, when taking school types into account, two different transition patterns occur. While longer periods of illness, unemployment and unstable employment contracts are reported from those attending regular schools, attendees of special schools describe their transition into vocational rehabilitation as comparably institutionalized. They learn about the possibility of vocational rehabilitation from career counselors of the LEA at school. The description of a 20-year old special school graduate illustrates the institutionalized transition process:

“I just got this letter from the Local Employment Agency telling me that I was transferring to a one-year pre-vocational program [Berufsvorbereitungsjahr] … I got a year’s training there as a metalworker. And after that, after the pre-vocational program, I got a letter again, this time inviting me to the next preparation program [berufsvorbereitende Bildungsmaßnahme = pre-vocational training].” (EEG_M_g)

Students attending special schools seem to be diagnosed with psychological disorders early in their life course. Among attendees of regular schools, psychological disorders often seem to occur later during school attendance and increase the chance of interruptions or dropouts from school, possibly due to a phase of disorientation. Attendees of regular schools seem to learn about vocational rehabilitation from others than professional career counselors, e.g., from medical professionals during hospitalization as a 25-year old woman who dropped out of university reports:

“At hospital I was shown various options of what I could do next and one of them was a vocational rehab program and I said that doesn’t sound bad. ... I’m glad that in the hospital they emphasized this opportunity” (EEG_L_a)

### Qualitative Results II: Rehabilitation Phase for Rehabilitants with Psychological Disabilities

In order to prepare for vocational training, specific preparation measures that combine medical and vocational rehabilitation are available for those with psychological disabilities. These measures take place in specialized institutions—known as RPK units.^4^ For those in need for these specific labor market programs, RPK units provide coping skills, occupational preparation (e.g., trying out potential occupations, training of everyday skills), training measures (e.g., training for and trying out a particular occupation before starting the vocational training as such), occupation-specific adaptive measures alongside psychological and medical treatment for up to 12 months. The interviewees describe the comprehensive support they receive as their best chance in order to subsequently obtain vocational training.

A female rehabilitant describes the wide range of therapies offered within a RPK unit. In addition to one-to-one sessions and group sessions, she was also offered practical training such as communication training (e.g., how to make telephone calls). She reflects:

“At that place there isn’t anything that’s compulsory, they simply offer you these different therapy options and they’re really useful, most of them are really useful if you actually use them. Yeah, there’s plenty on offer.” (EEG_L_a)

Besides psychological and medical stabilization and occupational orientation, preparation measures at RPK units allow for a gradual adaption to the schedule of working life. As the following quotation illustrates:

“At the beginning I had the problem, I’d not done anything for four years, and simply to get into the rhythm of work ... and so the way I got started was at first just going there in the morning until 12 and then I increased that, getting up to the whole day by the time I started my apprenticeship. And now the apprenticeship’s started it’s not a problem anymore.” “...the pre-vocational program was a great way of building up to a full daily routine and a full working day.” (EEG_M_d)

Since periods of illness, hospitalization and disorientation are described to be accompanied by a loss of social contacts and isolation, gradual adaption to interaction with others seem to further contribute to individual stabilization.

Furthermore, active participation in a working environment and the experience of success and improvement appear beneficial for recovery. Participation in vocational rehabilitation therefore seems to promote the sense of self-awareness and self-empowerment of young rehabilitants as the following quotation of the same interviewee illustrates:

“And in my case, I think things, they can only get better if I, if I do something. And that I manage to achieve something and somehow get back some self-confidence. And, in fact, that’s the only reason, I think, that I’ve got better.” (EEG_M_d)

After orientation and stabilization measures, often vocational training measures proceed in order to gain sustainable employment. As described above, these measures can be organized in a company-external or a regular in-company manner. Our sample is only comprised of individuals with company-external training measures.

“Completing ‘normal’ company-internal vocational training would possibly not be successful. … I would fail again. … I am not completely fit. … I would like to complete this vocational training [within the vocational training center].” (EEG_L_d)

This quotation of a 25-year-old man illustrates a high level of self-reflection on the own abilities and therefore the wish to attend a company-external training measure. Besides possible restrictions caused by the course of the disease, own abilities and personal preferences as well as the educational level or former school dropouts determine the choice for company-external vocational training.

Within the phase of vocational training the coping process continues as a 24-year old man describes:

“I was afraid of situations, in which I did not know what to expect. … I absolutely tried to settle every possible scenario. … This [behavioral pattern] slowly diminished. … As I am now heading towards job search and job interviews, I do not know [how this will be]; I can barely assess that myself, but at the moment during my vocational training I don’t have any problems, anymore.” (EEG_M_d)

In sum, rehabilitants report of the vocational rehabilitation as a phase that provides stabilization and job orientation.

## Discussion

In Germany, about one-fifth of all young rehabilitants are diagnosed with a psychological disability. The size of this group has been growing over recent years. Our study is the first to present a broad picture of vocational rehabilitation during the labor market entry phase of youth with psychological disabilities in Germany. The use of administrative data allows us to compare the population studied to young rehabilitants with other disabilities to point at special needs before, during and after the rehabilitation process. The administrative data also includes information on school and employment trajectories before vocational rehabilitation. Thus, we portray a complete picture of their way into vocational rehabilitation. In addition, enhancing the information available in the administrative data with qualitative interview data we are able to provide a deeper understanding of the individual ways into vocational rehabilitation and the motivation, self-assessment and challenges of youth with psychological disabilities during their labor market entry phase.

First, looking at the *school to work transition phase*, the results show that school to work transitions constitute a particularly vulnerable phase in the life course of youth with psychological disabilities. Although in general, school to work transitions become longer [33], those with psychological disabilities show even longer transition phases from school to work what goes along with a higher average age at the beginning of rehabilitation. Our quantitative results further show that the transition period is often associated with periods of unemployment, especially among young rehabilitants with psychological disabilities that are younger than 20 years. In sum, the quantitative findings indicate that individuals with psychological disabilities struggle more than other young adults with disabilities during their transition phase.

Accordingly, our results show that already at a very young age many rely on social benefit receipt. The qualitative interviews reveal many different obstacles these young men and women have to overcome, such as difficult family situations, serious struggles at school and lack of social as well as financial support. Also, it seems that in contrast to other disabilities, psychological disabilities often get diagnosed during the school to work transition phase. Therefore, further research should emphasize on the social situation of youth with psychological disabilities and test for causality between social circumstances and disability management.

Qualitative interviews also suggest that personal development of youth with psychological disabilities is hampered by the impairment and difficulties in finding a career perspective. In addition, the psychological disability aggravates their situation and psychological obstacles hinder finding a career. It takes time to recover or to learn how to cope with psychological issues. Therefore, a deeper perspective towards the relationship between psychological stability and social development during school to work transitions is necessary.

In this context, the qualitative interviews further suggest that there are at least two different patterns of transition. On the one hand, we can identify a group of students from special schools with gapless transitions from school to vocational rehabilitation. They represent a group who was affected by psychological disorders already earlier in their life course. This leads to special school attendance, close supervision and institutionalized intervention at the end of schooling, e.g., in early access to vocational rehabilitation. On the other hand, for students coming from mainstream schools or university with longer transition periods, phases of illness and recovery, late entries to vocational rehabilitation and school drop-outs can be observed. They tend to get diagnosed with a psychological disability at a higher age. Further research is necessary whether or not these two groups can also be identified in a representative sample in order to implement targeted support to provide early access to institutional support that can ease school to work transitions.

For instance, the qualitative interviews hint at problems in identifying rehabilitation needs possibly explaining the statistically identified longer transition rate from school to vocational rehabilitation. The FEA seems to have trouble in reaching youth with psychological disabilities, especially when they are visiting regular schools. However, it seems important to analyze how graduates and school drop-outs are supported in their transition phase and which special offers as well as “early warning systems” might help to reach out to those with psychological disabilities—at best before psychological disabilities manifest.

Second, with regard to *the rehabilitation phase*, the quantitative results show that youth with psychological disabilities remain longer in rehabilitation as well as in vocational education during rehabilitation than those with other disabilities (except for young adults with learning disabilities in vocational training). The qualitative interviews indicate that preparation and stabilization measures are necessary for those with psychological disabilities potentially prolonging the process of rehabilitation. In special treatment measures and company-external vocational rehabilitation, youth with psychological disabilities learn to adopt daily routines and to interact with customers and colleagues.

Finally, with regard to the *transition into employment* after completing vocational education, young men and women with psychological disabilities show lower transition rates into employment than those with other disabilities. Thus, on the one hand, a lack of appropriate jobs can be assumed. On the other hand, again a particularly vulnerable transition phase has to be overcome which is not necessarily successful.

In the overall discussion of our findings, the following limitations must be considered. First, although our quantitative analysis was conducted with a full census of administrative data, administrative records lack detailed information, e.g., on specific occupational restrictions due to the registered disability and on the complexity of health situations. Second, qualitative interviews were only conducted with a small sample of rehabilitants and, thus, analyses do not claim to be representative for the whole population studied. However, the complex realities of individuals with psychological disabilities can only be understood through individual perspectives. When sampling the interviewees, we followed the principle of contrast and chose interviewees with different characteristics with regard to gender, age, educational background and institutional setting [29]. Therefore, common experiences across different individuals can be identified. Finally, all rehabilitants in the qualitative sample were interviewed during program participation. Thus, no follow-up after program completion was conducted and, hence, no conclusions can be drawn on how program participation was assessed after completion and how the transition from vocational rehabilitation into the labor market is perceived. Accordingly, our results provide little evidence concerning the role of employers in the process of vocational rehabilitation of young people with psychological disabilities. These are topics for future research. Although our qualitative results suggest, that many obstacles to regular employment are rooted in psychological diseases, future research should also focus on further reasons. It needs to be analyzed to what extend the arrangements in company-external training contribute to lower transition rates in a way that their curriculum does not meet actual demands or that graduates from non-regular vocational training are stigmatized.

## Conclusion

The current study shows that among young persons with disabilities, those with psychological disabilities are particularly confronted with obstacles during their labor market transition. In Germany, vocational rehabilitation is meant to assist youth with disabilities in order to obtain vocational education and sustainable employment. Our exploratory approach sheds light on the growing group of young people in vocational rehabilitation with psychological disabilities. However, our results hint at access difficulties to vocational rehabilitation as well as difficulties during and after vocational rehabilitation among youth with psychological disabilities. Thus, recognition of psychological health problems needs to be improved and eligibility for vocational rehabilitation should be granted already in school. In addition, an overall system supporting school drop-outs and youth in early unemployment with psychological health problems could help to improve employment prospects of youth with psychological disabilities.

Furthermore, longer durations of rehabilitation and vocational education within rehabilitation might hint at further difficulties of youth with psychological disabilities. In this context, company-external vocational training including preparation and stabilization courses might prolong the process of rehabilitation. The higher share of rehabilitants with psychological disabilities quitting rehabilitation due to health issues further reflects the episodic character of psychological diseases [9]. Specific programs allowing to slowly adopt to regular employment should help to strengthen the workability of youth with psychological disabilities. However, lower transition rates into employment after rehabilitation seem to indicate that the disability-specific program cannot fully contribute to employability among the target group. Though, psychological stabilization is an important part of vocational rehabilitation, e.g., stepwise adaptation of a normally structured day, providing vocational orientation and perspectives. Still, the opportunity of company-internal phases during the training process should be increased even more in order to allow youth with psychological disabilities to adapt to regular work environments.

Finally, our results show that future qualitative and quantitative studies on vocational rehabilitation and the subsequent labor market transition process should take persons with psychological disabilities and their specific needs particularly into account. In this context, besides individual also external obstacles to employment, such as attitudes of employers towards people with (psychological) disabilities should be taken into account.

## Footnotes

1 Young people are defined here as belonging to the group of “people entering the labor market the first time” [*Ersteingliederung*]. This is a group administratively defined as being younger than 25 years and/or having low vocational experience of less than 3 years. People without a vocational degree are often also subsumed within this group.

2 In Germany, in 2013/14 two-thirds of school age children with disabilities still attend special schools [12]. However, following the ratification of the United Nations Convention on the Rights of Persons with Disabilities, inclusive education is on the increase. Instead of being educated in special schools, more and more children with disabilities are educated in mainstream schools. Therefore, also the way to identify and inform potential candidates about the possibility of vocational rehabilitation has to be further reorganized.

3 Usually interviews started with the following question: would you please tell me how you became a participant in this vocational training program/in vocational preparation etc.?

4 Attending a RPK unit is a measure for people with psychological disabilities in a specific institution within the context of vocational rehabilitation. The term RPK is based on the German term: “Rehabilitation psychisch Kranker”, i.e. rehabilitation of people with psychological illnesses.
